# Structural pharmacogenomics of drug-associated SNPs in oral squamous cell carcinoma

**DOI:** 10.3389/fgene.2026.1666142

**Published:** 2026-02-12

**Authors:** Punya Sunil, K. C. Ananth Kumar, A. I. Ananthakrishnan, Sebanti Gupta, Ranajit Das

**Affiliations:** Division of Data Analytics, Bioinformatics and Structural Biology (DABS), Yenepoya Research Centre, Yenepoya (Deemed to be University), Mangalore, Karnataka, India

**Keywords:** chemoresistance, DNA repair genes, oral squamous cell carcinoma, personalized medicine, pharmacogenomics, single-nucleotide polymorphisms

## Abstract

**Introduction:**

Pharmacogenomics enables the interpretation of how genetic variation influences drug response, offering a route toward precision oncology. Oral squamous cell carcinoma (OSCC) is a highly aggressive malignancy characterized by marked inter-individual variability in response to chemotherapy and radiotherapy, particularly in South Asian populations. However, the mechanism through which OSCC-associated genetic variants alter protein structure and drug interactions remains poorly understood.

**Methods:**

To address this, we integrated OSCC-specific variants from a Southern Indian patient cohort with pharmacogenomic annotations from ClinPGx. First, OSCC variants were mapped to drug-associated single-nucleotide proteins, yielding 22 protein-altering variants. Second, structural availability and functional relevance were used to prioritize eight variants for detailed analysis. Third, homology modeling and molecular docking were applied to evaluate how these variants influence protein conformation and drug binding.

**Result:**

This multistep pipeline identified variants in *FLT3, ATM, MUTYH, XRCC1, XPC*, and *MSH3* that affect DNA repair, signaling, and drug interaction interfaces. The highly prevalent *FLT3* T227M (rs1933437) variant was predicted to alter receptor dimerization and potentially modulate sunitinib binding. The *MUTYH* Q310H (rs3219489) variant, which is located near a zinc-binding motif in the interdomain connector, was predicted to perturb metal coordination and enzyme architecture, which indicates impaired base-excision repair.

**Conclusion:**

These findings demonstrate how pharmacogenomics-guided structural analysis can reveal mechanistic links between OSCC-associated variants and therapeutic response. While our results are based on *in silico* modeling, they provide a biologically grounded framework for prioritizing variants for experimental validation and for advancing population-specific precision oncology in OSCC.

## Introduction

Pharmacogenomics, the study of how genetic variations influence drug responses, is revolutionizing the field of medicine by enabling personalized treatment strategies tailored to an individual patient’s genetic profile (Evans and Relling, 1999; Stefanicka-Wojtas and Kurpas, 2023). This approach replaces the prevalent medical practice of the “one-size-fits-all” approach, optimizing therapeutic efficacy while minimizing the occurrence of adverse drug reactions (Wang and Wang, 2023). The integration of pharmacogenomics into clinical practice has demonstrated significant potential across a range of medical disciplines, particularly in oncology, where it addresses the complexities of cancer treatment and drug resistance mechanisms (Anghel et al., 2024; Crews et al., 2012).

Cancer, characterized by its genetic and molecular heterogeneity, is a prime target for pharmacogenomic applications. Variations such as single-nucleotide polymorphisms (SNPs), copy number alterations, and somatic mutations profoundly influence the efficacy of chemotherapeutic agents [cisplatin (Rocha et al., 2018)], targeted therapy [vemurafenib (Chapman et al., 2011)], and immunotherapy [pembrolizumab (Marcus et al., 2019; Konda et al., 2023)]. For example, polymorphisms in genes encoding drug-metabolizing enzymes such as CYP2D6, DPYD, and UGT1A1 are known to affect the metabolism of widely used anticancer agents tamoxifen, fluoropyrimidines, and irinotecan, respectively (Sánchez-Bayona et al., 2025; Varughese et al., 2020). Understanding how structural changes in mutated proteins affect their function enables clinicians to personalize drug selection and dosing, thus reducing toxicity and improving outcomes (Weinshilboum and Wang, 2017).

The integration of pharmacogenomics into cancer treatment is also transforming immunotherapy, particularly in the context of immune checkpoint inhibitors. Polymorphisms in immune regulatory genes, such as those encoding PD-L1 and CTLA-4, have been associated with variability in the response to checkpoint inhibitors such as pembrolizumab and ipilimumab (Buchbinder and Desai, 2016). These insights not only optimize patient selection but also pave the way for the development of combination therapies that enhance therapeutic efficacy.

Chemotherapy resistance is a formidable challenge in oncology, accounting for up to 90% of cancer-related deaths (Alfarouk et al., 2015). Both intrinsic and acquired resistance mechanisms, often driven by genetic and epigenetic alterations, contribute to treatment failure and disease recurrence (Dhanyamraju, 2024). For instance, mutations in the *EGFR* and *KRAS* genes are associated with resistance to tyrosine kinase inhibitors and cetuximab, respectively, highlighting the importance of genetic profiling in selecting appropriate targeted therapies (Li et al., 2018). Pharmacogenomic studies have also elucidated the role of the ABC transporter family in multidrug resistance, where overexpression of P-glycoprotein reduces the intracellular accumulation of chemotherapeutic agents (Gottesman et al., 2002). Addressing these resistance mechanisms through genetic insights has become a focal point of precision oncology.

Oral squamous cell carcinoma (OSCC), a subset of head and neck squamous cell carcinoma (HNSCC), underscores the critical need for implementing pharmacogenomic approaches in cancer. Representing over 90% of oral-cavity cancers, OSCC is the sixth- most common malignancy worldwide, with the annual incidence exceeding 500,000 cases (Giovannacci et al., 2016). OSCC, which accounts for more than 90% of malignancies of the oral cavity, represents a major clinical challenge due to late-stage diagnosis, aggressive local invasion, and frequent resistance to chemo-radiotherapy. OSCC is particularly prevalent in South and Southeast Asia, where the concurrent use of tobacco, smoking, alcohol, and betel nut acts synergistically with genetic susceptibility to drive tumorigenesis. Despite standardized treatment regimens combining surgery, radiotherapy, and platinum-based chemotherapy, patient outcomes remain highly variable, highlighting the need for genetically informed therapeutic stratification.

Recent genomic studies have identified recurrent variants in DNA repair and signaling genes such as *FLT3, XRCC1, XPC, MUTYH, ATM, ERCC5, EGFR,* and *MSH3* in OSCC patients (Paul et al., 2025), many of which are also implicated in drug sensitivity, toxicity, and resistance. Several of these genes interact directly or indirectly with widely used agents, including cisplatin, doxorubicin, sunitinib, and radiotherapy-induced DNA damage, making them particularly relevant for pharmacogenomic evaluation. Understanding how OSCC-specific variants alter the protein structure and drug-binding behavior is, therefore, essential for predicting the treatment response and minimizing toxicity in this malignancy.

To understand how cancer-associated SNPs contribute to disease onset and therapeutic resistance, it is essential to evaluate their impact at the protein structural and functional levels. Recent advancements in bioinformatics and structural biology have substantially improved our ability to analyze how genetic mutations influence drug-binding sites and protein stability, thereby regulating therapeutic outcomes (Ammar et al., 2023). Sequence- and structure-based databases, homology modeling (e.g., SWISS-MODEL), molecular docking, and molecular dynamics simulations facilitate the prediction of mutation-induced conformational changes that affect drug interactions and resistance (Konda et al., 2023). For example, identification of BRAF V600E mutations in melanoma led to the development of vemurafenib, demonstrating how structural pharmacogenomics can guide drug discovery (Chapman et al., 2011).

Despite its transformative potential, challenges still remain in incorporating pharmacogenomics into routine oncology practice. Variability in drug metabolism, limited access to genetic testing, and the need for robust clinical guidelines are significant barriers (Evans and Relling, 1999). However, ongoing research and technological advancements are addressing these limitations, fostering a future where cancer treatments are safer, more effective, and personalized to the genetic profiles of individual patients (Stefanicka-Wojtas and Kurpas, 2023).

To address the gap in OSCC-specific pharmacogenomic insights and understand how genetic alterations prevalent in the Southwest Indian population may influence therapeutic responses, we integrated mutational profiling of genes identified in a recent study on 33 oral cell carcinoma patients (Paul et al., 2025) with structural and drug-interaction analyses. By annotating clinically relevant SNPs with known chemotherapeutic associations and evaluating their potential impact on protein conformation and drug binding, this study aims to identify molecular determinants that may underlie treatment resistance or toxicity in OSCC. The aim of this study was to integrate OSCC-specific genetic variation with pharmacogenomic knowledge and structural modeling in order to identify drug-associated SNPs that may influence the protein function, treatment response, and therapeutic vulnerability in OSCC.

## Methods

**FIGURE 1:**
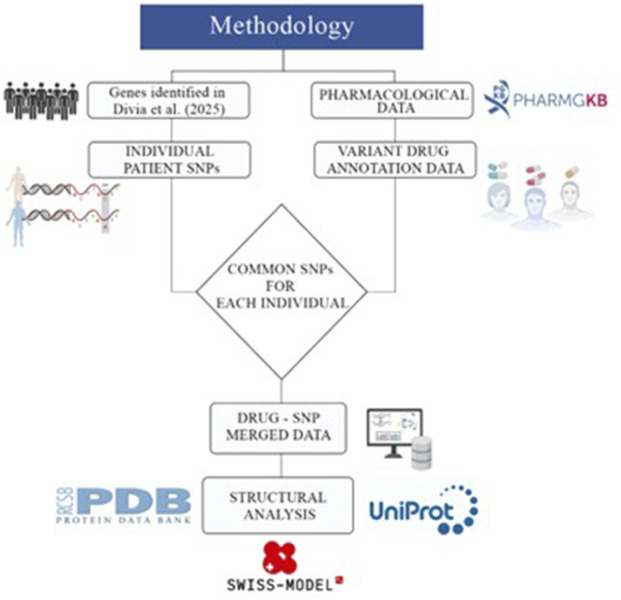
Summary of the methods used in this study.

We curated cancer-associated drug–variant annotations from ClinPGx and OSCC-specific variants from a Southern Indian patient cohort. These datasets were integrated to identify pharmacogenomically relevant SNPs in OSCC. The variants were then filtered based on functional and structural criteria to select proteins that are amenable to modeling. Finally, homology modeling and molecular docking were performed to evaluate how prioritized SNPs alter the protein structure and drug interactions. Figure 1 provides an overview of the integrated analytical workflow, illustrating the sequential steps from pharmacogenomic and OSCC variant curation through SNP filtering, protein structure modeling, and molecular docking to identify drug-associated structural effects in OSCC.

### Pharmacological and pharmacogenomics data Collection

Drug and variant data related to cancer were retrieved from the ClinPGx database (formerly PharmGKB, last accessed on 16th April 2025) (https://www.clinpgx.org) and utilized for further analysis. Similarly, data on chemotherapeutic drugs for cancer were curated. A total of 909 pharmacogenomic SNPs were identified as being associated with chemotherapeutic responses in cancer. The drug associations and their directions of effect were further obtained from ClinPGx, revealing that different drugs exhibit varying impacts on oral cancer. These 909 SNPs were found to be associated with 22 drugs based on ClinPGx annotations.

Here, we note that while the primary focus of this study is on OSCC biological mechanisms, ClinPGx curates pharmacogenomic associations across multiple cancer types and therapeutic contexts. Therefore, all cancer-related drug–variant annotations were considered during the initial data integration in order to capture variants with established effects on chemotherapy response, toxicity, or resistance that may also be relevant to OSCC. These multicancer annotations were subsequently mapped onto the OSCC-specific variant dataset derived from Paul et al. (2025), enabling disease-focused but pharmacogenomically comprehensive analysis.

### OSCC data curation

Genes and genetic variants identified from the OSCC cohort recruited from Southern India (Paul et al., 2025; data available at https://bcga.iitm.ac.in/cbioportal/study/summary?id=HNSCC_Yenepoya_2025) were annotated with drug–variant information using ClinPGx (Table 1). ClinPGx integrates pharmacogenomic evidence from multiple ethnicities and global populations, enabling comparison of locally observed OSCC variants with internationally curated drug-response data. Through this integration, 34 SNPs mapping to 28 pharmacogenomically relevant genes were identified as being associated with at least one chemotherapeutic agent, with annotations including drug response and direction of effect. These gene–drug–variant relationships form the basis for subsequent structural and functional analyses.

**TABLE 1:** Summary of genes identified from a Southern Indian OSCC cohort (Paul et al., 2025) that harbor SNPs associated with chemotherapeutic drug-response according to ClinPGx. For each gene, the corresponding drug(s) and associated SNPs are shown, representing the initial gene–drug–variant space from which the structurally analyzable SNPs were subsequently filtered.

Genes^a^	SNPs	Drugs
*EGFR*	rs2293347and rs2227983	Imatinib, cetuximab, EGFR inhibitors, fluorouracil, and gefitinib
*TP53*	rs1042522	Platinum compounds, antineoplastic agents, capecitabine, paclitaxel, cisplatin, and oxaliplatin
*ERCC5*	rs1047768and rs17655	Fluorouracil, platinum compounds, radiotherapy, and platinum
*ERCC2*	rs1799793	Bevacizumab, bleomycin, capecitabine, cisplatin, docetaxel, epirubicin, etoposide, fluorouracil, gemcitabine, oxaliplatin, platinum compounds, radiotherapy, and trastuzumab
*DHFR/MSH3*	rs1650697	Pemetrexed
*RET*	rs1799939	Imatinib
*ATM*	rs1801516	Cyclophosphamide, doxorubicin, and fluorouracil
*KDR*	rs1870377 and rs2305948	Cisplatin, fluorouracil, oxaliplatin, imatinib, sorafenib, and sunitinib
*FLT3*	rs1933437	Imatinib and sunitinib
*XPC*	rs2228000 and rs2228001	Platinum compounds
*ERBB2*	rs1136201	Carboplatin, docetaxel, and trastuzumab
*ERBB3*	rs773123	Carboplatin, docetaxel, and trastuzumab
*XRCC1*	rs1799782 and rs25487	Carboplatin, cisplatin, cyclophosphamide, doxorubicin, fluorouracil, irinotecan, leucovorin, oxaliplatin, platinum, platinum compounds, and radiotherapy
*MUTYH*	rs3219489	Cisplatin, fluorouracil, and radiotherapy
*FGFR4*	rs351855	Cyclophosphamide, fluorouracil, methotrexate, and everolimus
*PDGFRA*	rs35597368	Imatinib
*FLT1*	rs7993418	Cisplatin, fluorouracil, oxaliplatin, and imatinib

^a^ Genes were curated from Paul et al.(2025) (data available at https://bcga.iitm.ac.in/cbioportal/study/summary?id=HNSCC_Yenepoya_2025) and annotated with drug-variant information from ClinPGx.

### SNP data curation

Data for each SNP were retrieved from the dbSNP web server (https://www.ncbi.nlm.nih.gov/snp/). The reference and alternative alleles were noted. The wild-type and mutant amino acids associated with each SNP were also recorded and used for downstream protein structural analysis.

### Screening of proteins based on structural and functional criteria

Genes identified in the Southern Indian OSCC cohort (Paul et al., 2025) were systematically filtered based on the mutation type, prevalence, and structural or functional relevance. These genes and their associated variants were then cross-referenced with ClinPGx to identify SNPs with documented or predicted associations to chemotherapeutic drug responses. This integration yielded 34 pharmacogenomically relevant SNPs distributed across 28 genes. Variants resulting in synonymous substitutions or noncoding transcript changes (12 SNPs) were excluded from structural analyses, leaving 22 protein-altering SNPs for downstream analysis, as summarized in Table 1.

### Structural and functional evaluation of the selected SNPs

The SNP set was narrowed from 22 to 8 based on the availability of experimentally determined protein structures in the RCSB Protein Data Bank (PDB) and UniProt structural repositories, which were required for reliable downstream modeling and docking. These eight SNPs were thoroughly analyzed using UniProt (The UniProt Consortium, 2025), RCSB PDB (Burley et al., 2025; RCSB, 2025), and dbSNP (Sherry et al., 2001) for the identification of the mutations present, the potential functional effects on cancer biology, the prevalence of the particular mutation in cancer, and finally, their structural characterization. Varying effects of these mutations in different populations were further evaluated from the existing literature.

Table 2 summarizes the eight OSCC-associated genes and variants selected for detailed structural analysis after ClinPGx-based filtering. For each entry, the table lists the corresponding SNP (rsID), amino-acid substitution, UniProt accession, availability of experimental or modeled protein structures, and the source references used to support the functional and structural relevance. These data were compiled from UniProt, RCSB Protein Data Bank (PDB), dbSNP, and published literature and served as the basis for selecting variants that are suitable for homology modeling, docking, and structural interpretation.

**TABLE 2:** Structural and functional annotation of the eight OSCC-associated variants selected for downstream modeling and docking. For each gene, the corresponding SNP (rsID), amino-acid substitution, and PDB ID are shown. References indicate the primary literature that determined the structure of these proteins experimentally.

Protein	PDB ID	Variant (rsID)	Amino acid substitution	References
ATM	7SIC	rs1801516	D1853N	Warren and Pavletich (2022)
XRCC1	2D8M	rs1799782 and rs25487	R194W and Q399R	Nagashima et al. (2006)
FLT3	3QS7	rs1933437	T227M	Verstraete et al. (2011)
MUTYH	8FAY	rs3219489	Q310H	Trasvina-Arenas et al. (2024)
XPC	8EBU	rs2228000 and rs2228001	A499V and Q939K	Kim et al. (2023)
MSH3	8OLX	rs1650697	I79V	Lee et al. (2024)

### Structural modeling and interaction visualization

Because MUTYH plays a critical role in base excision repair through a zinc-dependent inter-domain connector that stabilizes DNA binding and enzymatic activity, we specifically examined how the OSCC-associated Q310H substitution (rs3219489) might perturb zinc coordination and protein architecture. To study the altered networking of zinc in the mutated MUTYH protein, mouse MUTYH is used as a template to generate the structure of human MUTYH by using the SWISS-MODEL (Waterhouse et al., 2018) server in the presence of the Zn coordinate. The docking of FLT3 and sunitinib was performed to generate a representative binding model for the visualization of protein–ligand interaction on AutoDock (Morris et al., 2009) using different docking parameters (Kumar et al., 2025). Among the drug–protein interaction complexes generated, the complex that best reproduced the previously reported interaction (Acharya et al., 2022) pattern details was selected. The final model-figures were generated using PyMOL software (Schrödinger, 2023).

## Results and discussion

### Analysis of genes and genetic variants

The variants discussed in this section represent the OSCC-associated SNPs derived from the Southern Indian cohort of Paul et al. (2025) that were retained after cross-referencing with ClinPGx to identify pharmacogenomically annotated drug-response variants. We shortlisted the SNPs that are present in at least 70% of the recruited patients. These mutations were further assessed to explore their implications in cancer. Importantly, all the SNPs described below are those with documented or predicted drug-response associations in PharmGKB rather than the complete set of variants detected in the OSCC cohort.

The SNP rs7993418, which is present among all recruited patients and is located in exon 28 of the *VEGFR1* gene, has previously been associated with progression-free survival (PFS) in metastatic renal cell carcinoma (mRCC) patients undergoing bevacizumab treatment. This synonymous SNP affects the tyrosine 1,213 residue in the VEGFR1 tyrosine-kinase domain, altering codon usage and increasing *VEGFR1* expression and signaling. Studies, including the AVOREN trial, demonstrated a correlation between rs7993418 genotypes and treatment outcomes, with patients harboring the TT genotype showing the longest PFS. This SNP’s predictive value underscores its potential as a biomarker for guiding anti-angiogenic therapy in mRCC patients. Furthermore, the rs7993418 SNP (Tyr1213Ter) leads to a shift in codon usage, enhancing *VEGFR1* expression and downstream signaling, which is associated with a higher risk of disease progression and increased mortality (Beuselinck et al., 2014; Claes et al., 2012; Pallaud et al., 2014).

The *CCND1* gene, which regulates the normal cell cycle, was also implicated, particularly the rs9344 polymorphism located at the exon 4–intron boundary. This SNP has been linked to various cancers, including cervical, prostate, colorectal, and head and neck squamous cell carcinoma. The *CCND1* G870A polymorphism (rs9344) has shown potential as a prognostic biomarker for cancer, especially in the Indian population, where it may help predict the risk of cancers such as breast, esophageal, and colorectal cancer (Thakur et al., 2018).

Another significant SNP, rs1933437, has been associated with toxicity in patients treated with sunitinib, highlighting its potential to influence treatment outcomes (Alarcón-Payer et al., 2022). The *DHFR* gene, with its rs1650697 (G>A) variant, has also been identified as a favorable prognostic marker in non-small-cell lung cancer (NSCLC), indicating its role in risk stratification and personalized treatment (Jin et al., 2010; Xu et al., 2015).

The rs2228001 SNP in the *XPC* gene and, specifically, the AC/CC variants were significantly associated with skin dermatitis and elevated C-reactive protein (CRP) levels, indicating that mutations in *XPC* impair nucleotide excision repair mechanisms and increase the risk of severe skin reactions and systemic inflammation (Maćkowiak et al., 2024).

The *ERCC1* rs11615 polymorphism was significantly associated with an increased risk of breast cancer, particularly in Asian populations, underlining its potential role as a risk factor for breast cancer. Additionally, the synonymous rs7121 SNP in the *GNAS* gene, which plays a role in G-protein receptor signaling, was found to have implications for tumor progression and prolonged survival, thus showing promise as a biomarker for predicting therapy response (Li et al., 2018; Möhlendick et al., 2019).

High *XRCC1* expression, which is associated with poor overall survival and minor treatment response, was linked to the rs25487 polymorphism. This variant was associated with a reduced risk of minor treatment responses in esophageal cancer and an increased risk of high-grade side effects in head and neck cancer (Gong et al., 2021; Xu et al., 2018).

### Structural analysis

The proteins housing the 22 extracted SNPs (see Methods) are either involved in DNA repairing; cell proliferation, migration, and survival; or in cell signaling. While most of them have already been identified to be involved in different human carcinoma (McDuff et al., 2021; Narita et al., 2009; Sahu et al., 2016; Xiao et al., 2017), only a handful of these, namely, *MUTYH* [rs3219489 (Q310H)] (Senghore et al., 2020), *XRCC1* [rs1799782 (R194W); rs25487 (Q399R)] (Senghore et al., 2020), *ERCC5* [rs1047768 (H46Q); rs17655 (D1104H)] (Zuo et al., 2022), *EGFR* [rs2293347 (D994E); rs2227983 (R521K)] (Saravani et al., 2020), *XPC* [rs2228000 (A499V); rs2228001 (Q939K)] (Nigam et al., 2019), and *FGFR4* [rs351855 (G388R)] (Chou et al., 2017), have been reported to be associated with OSCC. In the current study, for the first time, we report variants such as *DHFR/MSH3* [rs1650697 (I79V)], *ATM* [rs1801516 (D1853N)], *FLT3* [rs1933437 (T227M)], *ERBB2* [rs1136201 (I655V)], *ERCC2* [rs1799793 (D312N)], *RET* [rs1799939 (G691S)], *KDR* [rs1870377 (Q472H) and rs2305948 (V297I)], *PDGFRA* [rs35597368 (S478P)], *ERBB3* [rs773123 (S1119C)], and *FLT1*[rs7993418 (T1213Stop)] from the southwest coast of India. Among them, eight SNPs were selected for further downstream analysis (Table 3C), and the remaining could not be utilized because of the unavailability of their structural details.

**TABLE 3:** A. Genes identified based on similarities between that curated from Paul et al. (2025) and from the ClinPGx database. B. Genes filtered in after excluding genes with synonymous substitutions and non-coding variants. C. Genes selected for structural and mutational analysis based on the availability of corresponding protein structures.

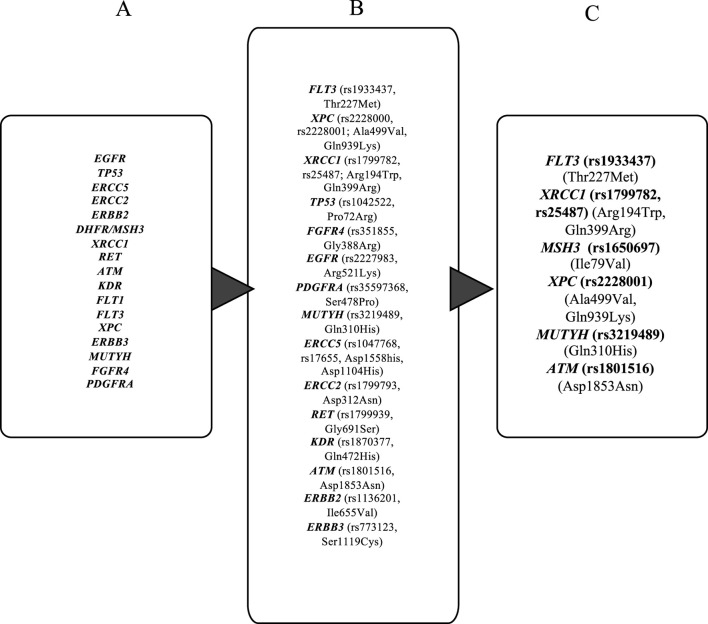

Table 4 summarizes the prevalence of the eight selected SNPs among the patients, their global and South Asian allele frequencies, and the reported clinical relevance in cancer. Several variants such as *FLT3* T227M*, XRCC1* Q399R*, MSH3* I79V, and *XPC* Q939K were highly prevalent (>80%) in our study population and occur at notably higher frequencies in South Asian populations compared to global averages, underscoring potential ancestry-specific pharmacogenomic implications. Most of these SNPs are located in genes involved in DNA repair and cell-cycle regulation, with prior evidence linking them to carcinogenesis, treatment response, or radiation and chemotherapy toxicity. These mutations reinforce the importance of population-specific genetic profiling to better understand therapeutic outcomes and optimize personalized treatment strategies for OSCC patients.

**TABLE 4:** Comparison of minor allele frequencies (MAF) of the eight SNPs utilized for structure–function association analysis among global datasets (https://gnomad.broadinstitute.org).

Gene	Variant (rsID; AA change)	Percentage of recruited patients with this mutation	gnomAD global MAF (approx.)	South Asian MAF (approx.; gnomAD/1000G/IndiGen)	Cancer/Clinical associations (concise)
*FLT3*	rs1933437 (T227M)	>80%	∼0.52 (alt allele) – common missense polymorphism in *FLT3*	∼0.65–0.70 (IndiGen ∼0.675; higher than global)	Germline missense in FLT3 (oncogenic kinase). Reported association with sunitinib toxicity and other TKI-related adverse effects; considered a pharmacogenomic variant in Indian cohorts. Additionally, it appears in GWAS signals linked to blood pressure and Tourette’s, but is not a classic high-penetrance cancer driver.
*XRCC1*	rs1799782 (R194W)	∼5%	∼0.05–0.10 globally (low-frequency; varies widely by ancestry, but consistently >0.05 in many datasets)	Variable; ∼0.10–0.25 overall, with reports from ∼0.09 in some North Indian groups to >0.25–0.30	Base-excision repair variant. Repeatedly studied in lung, colorectal, breast, and head and neck cancers with mixed but suggestive associations with risk. Several studies (including OSCC) indicate impact on response/toxicity to platinum chemo-radiotherapy via altered BER capacity.
*XRCC1*	rs25487 (Q399R)	>80%	Alt allele AF ∼0.65–0.70 in gnomAD/ExAC (very common)	South Asian MAF typically ∼0.35–0.45 (e.g., A-allele ∼0.42 in the South Indian cohort)	One of the best-studied DNA-repair SNPs. Associated (sometimes inconsistently) with risk of lung, breast, colorectal, ovarian, OSCC, and others and with platinum and cyclophosphamide efficacy/toxicity (e.g., better or worse response depending on the genotype). Frequently appears in pharmacogenomic panels for platinum therapy.
*DHFR/MSH3*	rs1650697 (I79V)	>80%	Global MAF ≈0.23 (1000G; “T”/variant allele) – common coding variant	SAS MAF ≈0.32 (1000G South Asian; variant allele)	Located in the DHFR/MSH3 locus (5′ UTR in DHFR, missense I79V in MSH3). Acts as an eQTL for MSH3 and DHFR and modifies somatic repeat instability and age at onset in Huntington’s disease and myotonic dystrophy. Additionally, it is studied as a predictor of platinum chemotherapy response via MSH3-mediated mismatch repair.
*XPC*	rs2228000 (A499V)	∼30%	Global MAF ∼0.20–0.25 (common; gnomAD reports frequency >0.01, Japanese ≈0.23)	Likely in similar range (∼0.20–0.30) in SAS but with inter-study variation (exact gnomAD SAS value depends on release)	NER gene variant. Reported associations with breast cancer, lung cancer, and other solid tumors, especially in Asian cohorts, often in combination with rs2228001. Generally considered a low-penetrance cancer risk modifier affecting nucleotide-excision repair efficiency.
*XPC*	rs2228001 (Q939K)	>80%	Global MAF ∼0.30–0.40; Japanese frequency ∼0.60 for the K allele, indicating it is common worldwide	SAS MAF probably ∼0.30–0.40 (common; precise value varies by cohort)	Another XPC missense variant; associated with risk of prostate cancer, bladder cancer, and other tumors, particularly in Asian populations, and sometimes with altered DNA repair capacity or therapeutic outcomes. Often analyzed in haplotype with rs2228000 in NER-cancer association studies.
*MUTYH*	rs3219489 (Q310H)	∼40%	gnomAD/ExAC global AF ≈0.25–0.27 (common missense polymorphism)	SAS MAF around 0.30–0.35 in several cohorts (e.g., supplementary tables report ∼0.30–0.38)	Common BER gene variant. ClinVar usually classifies it as benign for classic MUTYH-associated polyposis, but multiple studies link it to modified risk for colorectal, rectal, lung, and other cancers and to outcomes in OSCC chemo-radiotherapy. Best seen as a low-effect risk/response modifier and not a high-penetrance pathogenic mutation.
*ATM*	rs1801516 (D1853N)	∼20%	gnomAD freq ≈0.11 (alt allele)	South Asian MAF in 1000G ≈ 0.14; some South Asian cohorts (e.g., Sri Lankan) report higher variant allele freq (∼0.42), showing strong population heterogeneity	Key radiogenomic SNP in ATM. Numerous studies/meta-analyses link the A (Asn) allele with increased risk of late radiation toxicity (fibrosis, telangiectasia, pneumonitis, and rectal toxicity) after radiotherapy for breast, lung, prostate, and other cancers. The overall association with cancer risk *per se* is weak/inconsistent, but some data suggest reduced breast cancer risk for the AA genotype. Additionally, it appears in pharmacogenomic frameworks as a marker of chemo- and radio-toxicity (e.g., with cyclophosphamide/doxorubicin/5-FU).

Complete docking results, including the full AutoDock 4.2 log file (random seeds, scoring functions, binding energies, RMSD clustering, and ranked poses) for FLT3 and sunitinib, are provided in Supplementary Table.

### Structure–function correlation of cancer onset

**FIGURE 2:**
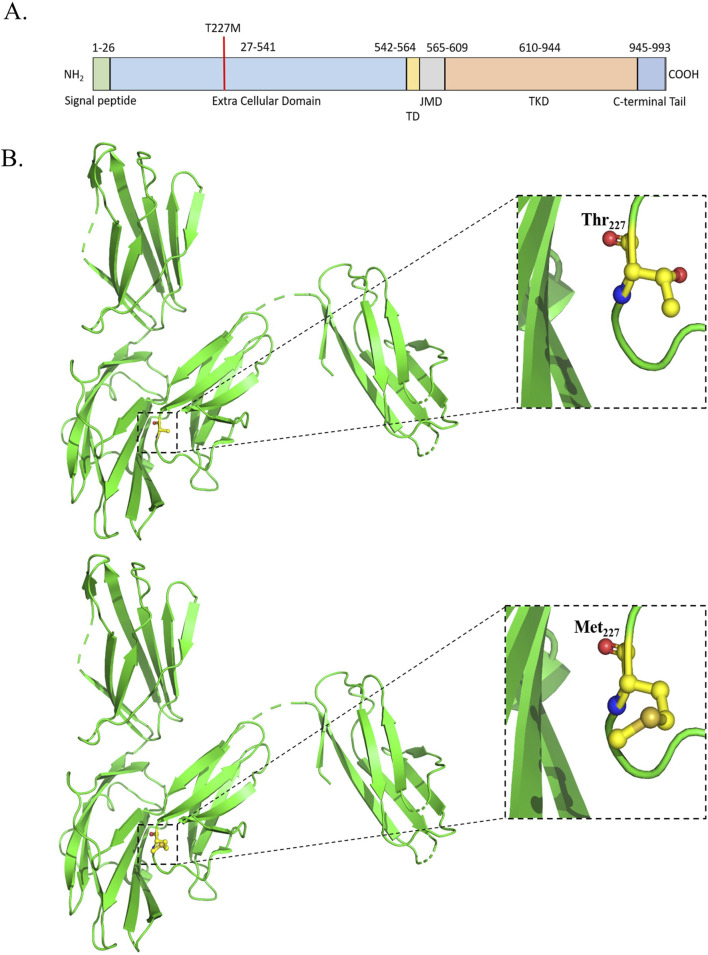
**(A)** Schematic representation of the domains and mutation of the human FLT3. **(B)** Structural representation of the mutation.

Fms-like tyrosine kinase receptor 3 [FLT3] (PDB ID: 3QS7) carrying T227M (rs1933437) alteration is identified among 80% of the OSCC patients. FLT3 regulates the proliferation, differentiation, and survival of hematopoietic progenitor cells (Griffith et al., 2004; Kazi and Rönnstrand, 2019). FLT3 is 993 amino acids long and consists of one extracellular domain [27–541] with five Ig-like subdomains [D1–D5], one transmembrane domain [542–564], one juxta membrane domain [565–609], and two tyrosine kinase domains[TKD] [610–944], followed by a C-terminal tail [945–993] (Kazi and Rönnstrand, 2019; Verstraete et al., 2011). Interaction with the FLT3 ligand causes dimerization of the protein, which triggers autophosphorylation at the cytosolic domains followed by downstream signaling (Griffith et al., 2004). Most of the carcinogenic mutations in FLT3 cause activation of TKD, resulting in aberrant proliferation 63 (Daver et al., 2019; Griffith et al., 2004; Rataj et al., 2025; Tecik and Adan, 2022). The T227M alteration in FLT3 is being isolated from OSCC patients for the first time (Figure 2). The D2 subdomain of FLT3 accommodates the 227 substitution, which is in close proximity to the interaction plane between D3 and FL. This may affect its interaction between D2 and D3 and alter the affinity for FL.

**FIGURE 3:**
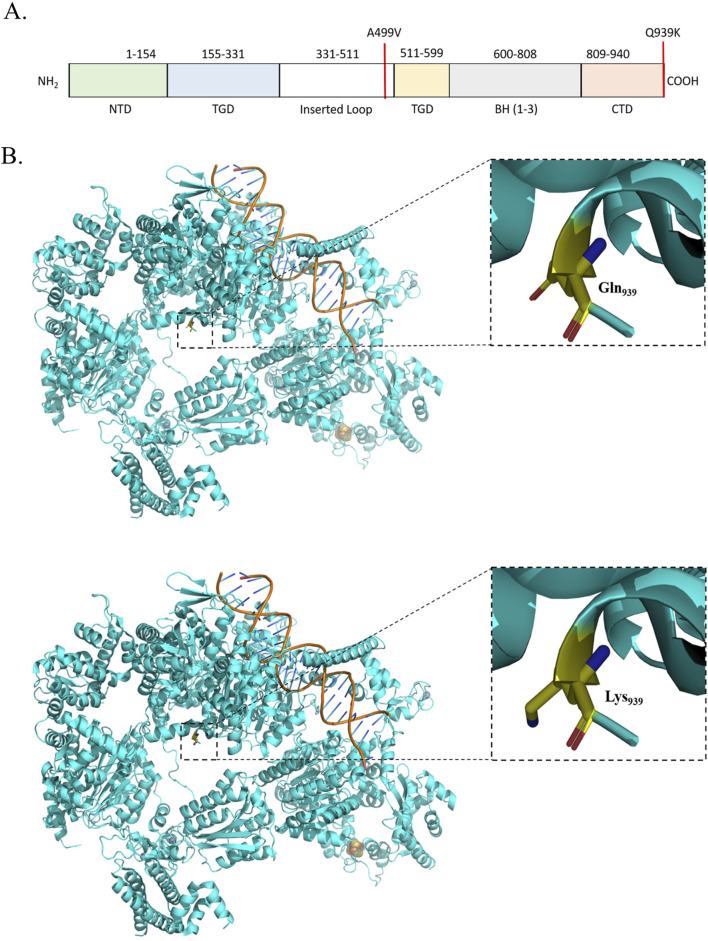
**(A)** Schematic representation of the domains and mutations of the human XPC. **(B)** Structural representation of the mutation in the XPC complex.

The Xeroderma pigmentosum complementation group C (XPC) (PDB ID: 8EBU) with either A499V (rs2228000) or Q939K (rs2228001) is distinguished for 30%–80% patient pool. XPC plays a crucial role in nucleotide excision repair [NER],base excision repair [BER], and in transcriptional regulation (Bunick et al., 2006). It contains 940 residues with the transglutaminase homology domain (TGD) and 3 β-hairpin domains 1–3 (BH) (Zhang et al., 2015). While XPC uses its TGD domain for communication with RAD23B, it retains its contact with CETN2 through BH domains (Kim et al., 2023; Zhang et al., 2015). The transcription regulation is catalyzed by XPC in complex with the seven-subunit TFIIH core complex (Core-7; seven subunits, namely, XPB, XPD, p62, p52, p44, p34, and p8). Two long helices, long helix N-terminal [LHN] spanning amino acid (aa) 167–224 and long helix C-terminal [LHC] spanning aa 815–866, which are 45Å apart, extend from XPC toward the TFIIH core. LHN contacts with the p62 domain of Core-7 when LHC ensures the stability of the complex with XPB. The subsequent three short helices L, M, and N encompassing the last 50 aa remain involved with XPB, p52, p8, and p34 (Gaur et al., 2018; Kim et al., 2023; Zhang et al., 2015). A499V mutation affects the XPC-Rad23 interaction, reducing DNA repair efficiency, and it has been reported with a higher risk of developing acute myeloid leukemia, bladder, and breast cancer (He et al., 2013; Lakkireddy et al., 2021) (Figure 3). Q939 caps the C-terminal helix of XPC and is important for communication with Core-7. Polymorphic variation at the 939 position has been reported to be associated with bladder, lung, and colorectal cancer (He et al., 2013; Lakkireddy et al., 2021; Sankhwar et al., 2016).

**FIGURE 4:**
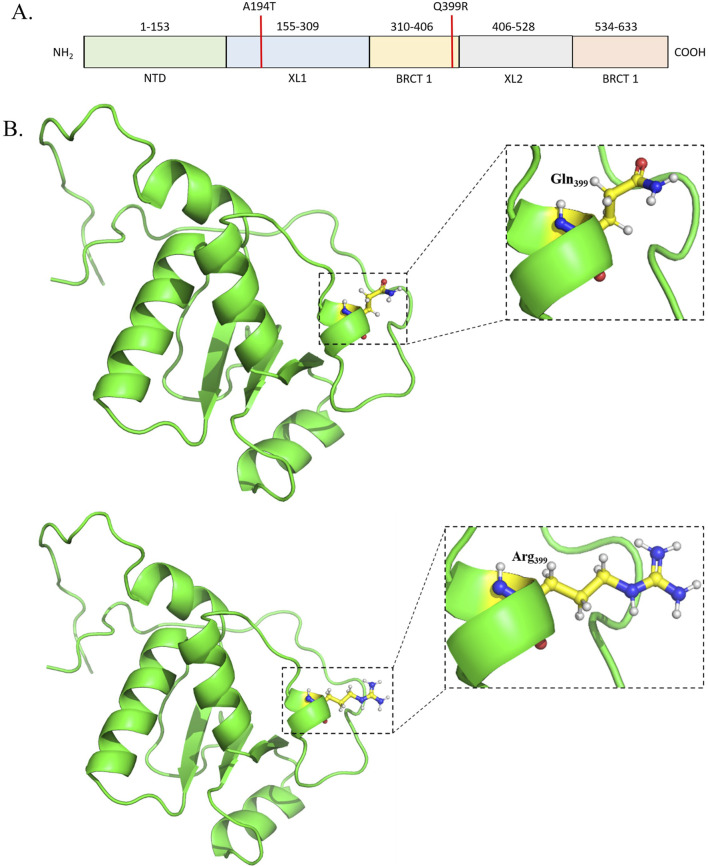
**(A)** Schematic representation of the domains and mutations of the human XRCC1. **(B)** Structural representation of the mutation.

**FIGURE 5:**
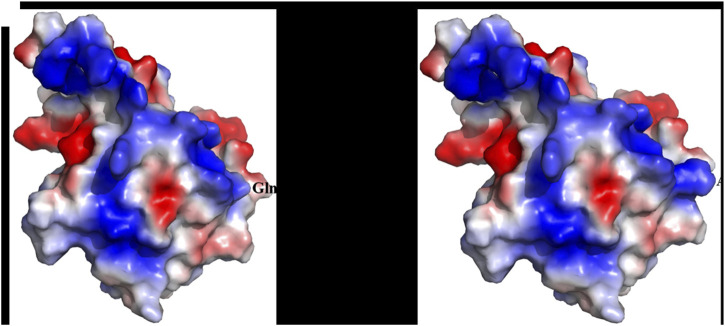
Electrostatic surface representation highlighting the positively charged region.

**FIGURE 6:**
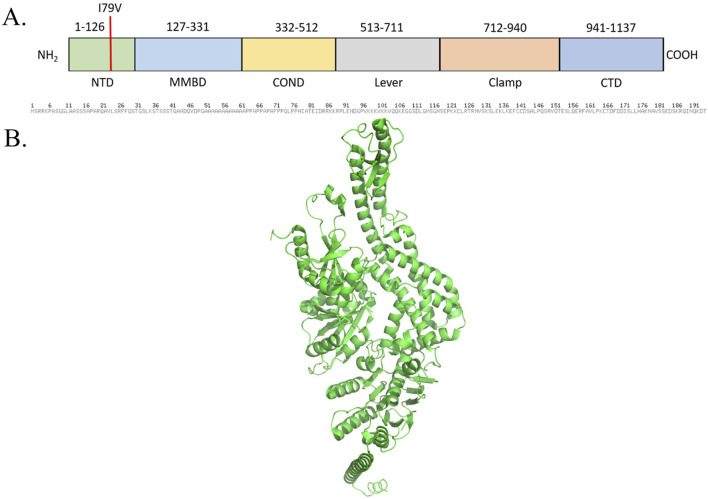
**(A)** Schematic representation of the domains and mutation of the human XRCC1. **(B)** Structural representation of the solved structure.

X-ray cross-complementing group 1 (XRCC1) (PDB ID: 2D8M) accommodating R194W (rs1799782) or Q399R (rs25487) correlates with 5%–80% OSCC patients. XRCC1 functions to repair the single-stranded DNA nicks in complexes with DNA ligase III, DNA polymerase b (b-Pol), and poly(ADP-ribose) polymerase (PARP) (London, 2015; Marintchev et al., 1999). XRCC1 is 633-aa-long and consists of three major domains, namely, N-terminal domain [NTD (1–183)], central BRCT domain [BRCT I (310–406)], and C-terminal BRCT domain [BRCT II (534–633)]. NTD retains a beta sandwich structure, facilitating interactions with DNA (Marintchev et al., 1999; Mok et al., 2019). BRCT I facilitates the recruitment of XRCC1 to the DNA damage site through the interaction with PARP1 while BRCT II remains in communication with DNA ligase III to promote the final DNA end ligation step (Caldecott, 2019; London, 2015; Marintchev et al., 1999; Zhang et al., 1998). These functional domains are interconnected via two linkers, XL1 [155 to 309] and XL2 [406 to 528]. R194T occurs due to a C to T substitution in exon 6. This region of the protein is crucial for maintaining genomic integrity and the BER pathway through coordination with PCNA-like proteins (Hu et al., 2005). Hence, this substitution can lead to reduced efficacy of DNA repair, increased susceptibility to oxidative stress, and finally, increased risk of cancer (Hsueh et al., 2017) (Figures 4, 5). Notably, according to most of the publications until now, the R to T substitution at 194 is more protective than the wild-type Arg/Arg genotype. This mutation occurs in the BRCT 1/a domain and is reported with reduced DNA repair efficacy (Matsuo et al., 2004). The structural analysis of the BRCT domain exhibits high density of the positively charged population in the region close to 399, and Q-to-R substitution will increase the degree further (Figure 6). As expected, homozygous Arg/Arg or heterozygous Arg/Gln has been found to have a significant correlation with cancer onset (Saad et al., 2021).

The MutS homologs (MSH) (PDB ID: 8OLX) are members of the DNA mismatch repair system, and the Msh2–Msh6 and Msh2–Msh3 forms are reported in humans (Pal et al., 2020). Human MSH3 is a 1,137-aa-long multi-domain protein. The N-terminal domain remained structurally unsolved yet, while the mismatch binding domain [MMBD] played the leading role in the identification of insertion–deletion loops [IDLs]. The clamp domain is responsible for DNA-binding, and two connector domains serve to link the DNA binding and ATPase domains. The C-terminal dimerization domain (DMD) ensures the stability of the heterodimer complex. Specific structural bending is triggered on IDLs by MSH3 (Gupta et al., 2011). The I 79 is located in the NTD and is important for crosstalk with EXO1, the master of DNA mismatch repair, which corresponds to 80% of patients with OSCC. In consequence, the I79V substitution (rs1650697) is found to be associated with increased risk of several types of cancers (Nam et al., 2021).

**FIGURE 7:**
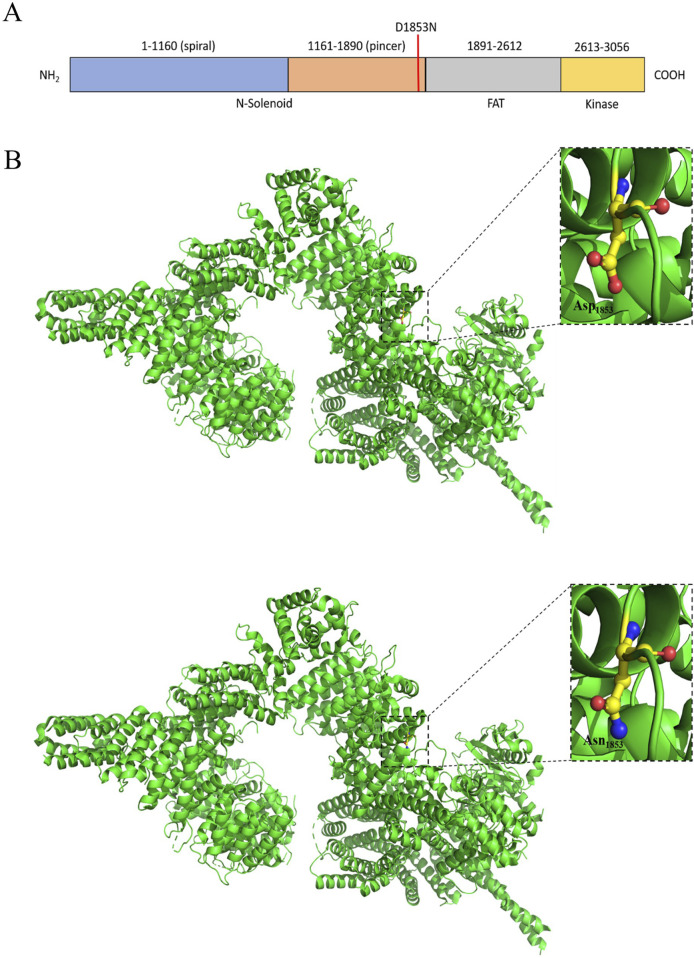
**(A)** Schematic representation of the domains and mutation of the human ATM. **(B)** Structural representation of the mutation.

The ataxia–telangiectasia mutated (ATM) (PDB ID: 7SIC) is a protein kinase that functions as a master regulator of the DNA damage response (García et al., 2022; Phan and Rezaeian, 2021; Ueno et al., 2022). Inactivated ATM remains in the butterfly-shaped dimeric state, and on interaction with the Mre11–Rad50–Nbs1 (MRN) complex due to DNA breaks or oxidation, it retains the monomeric status (Baretić et al., 2017; Warren and Pavletich, 2022). ATM is a multi-domain protein of molecular weight 350 KDa with 3,056 aa, which can be divided into the N-solenoid and FATKIN regions. N-solenoid comprises the spiral (residues 1–1,166) and pincer (1,167–1,898) domains with multiple HEAT repeats. The residues from 90 to 97 in the spiral domain play an important role in the interaction with p53, BRCA1, and LKB1. Nbs1 of the MRN complex binds at the interface between the spiral and pincer domains (Baretić et al., 2017; Warren and Pavletich, 2022). The FATKIN consists of FAT (1,899–2,613), kinase domain (2,614–3,026), and FATC (3,027–3,056) domains. Three tetratricopeptide repeat domains TRD1 (1,899–2,025), TRD2 (2,026–2,192), TRD3 (2,193–2,479), and HRD (2,480–2,613) subdomains encompass the FAT region, while the N-lobe (2,614–2,770), C-lobe (2,771–2,957), and PRD (2,958–3,026) subdomains add up to the kinase domain (Figure 7). The FAT domain is responsible for ATM dimerization, while autophosphorylation on S1981 at TRD1 is reported to be pivotal for the monomeric activation status of the ATM (Bakkenist and Kastan, 2003). Once activated, ATM associates with the phosphorylation of downstream targets that involves approximately 905 phosphorylation sites over 700 proteins to initiate a signaling cascade through the HR pathway (García et al., 2022; Matsuoka et al., 2007). D1853N substitution has been identified for OSCC for the first time in India. D1853N (rs1801516) is present at the pincer domain, which is the binding site of the MRN complex. As expected, this alteration has been reported to be associated with cancer (Li et al., 2009).

**FIGURE 8:**
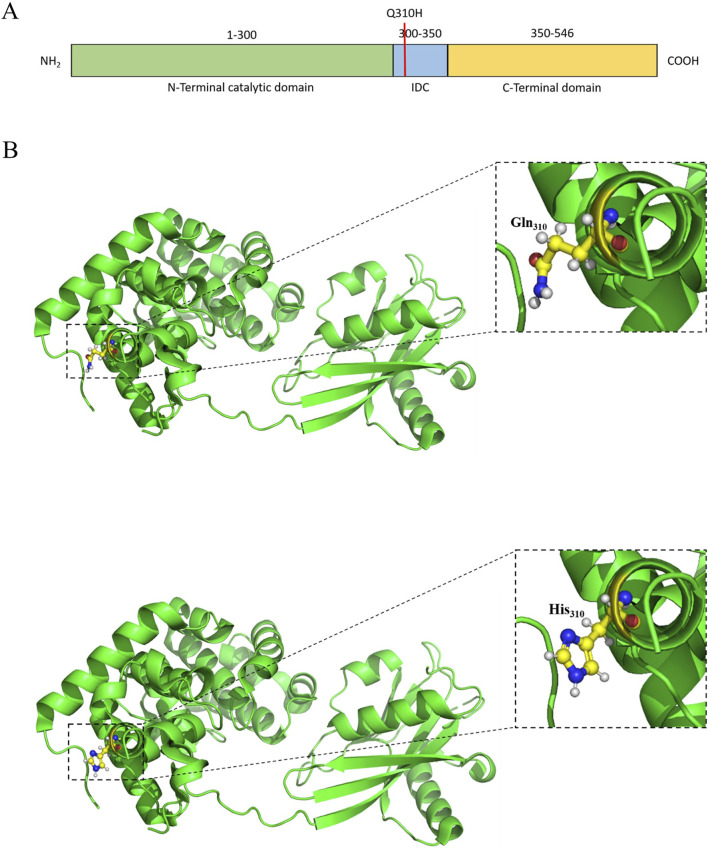
**(A)** Schematic representation of the domains and mutation of the human MUTYH. **(B)** Structural representation of the mutation.

**FIGURE 9:**
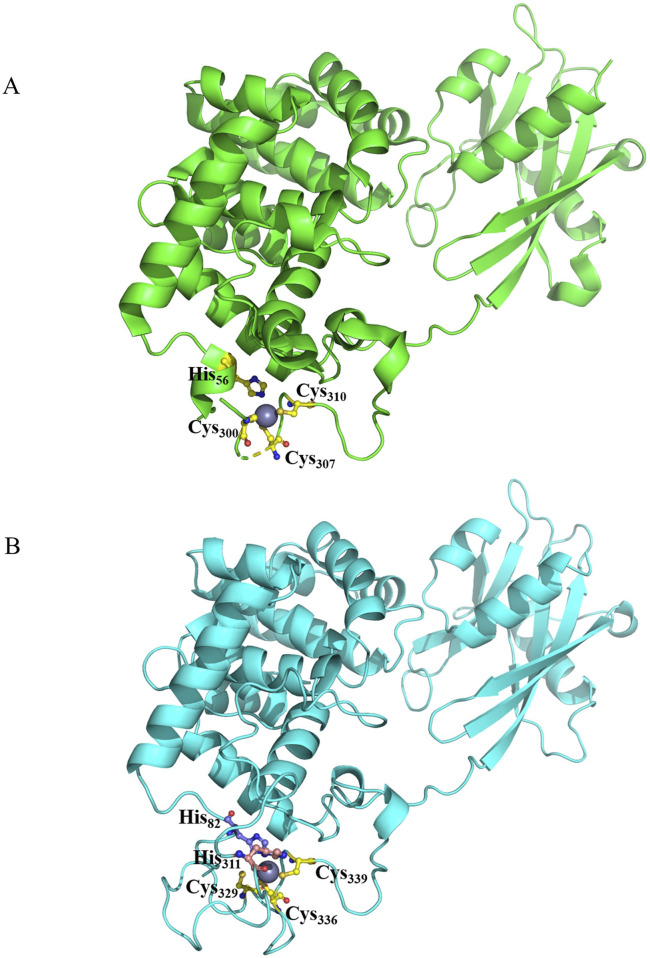
**(A)** Structural representation of mouse MUTYH highlighting residues interacting with Zn ion. **(B)** Structural representation of human MUTYH highlighting residues interacting with Zn ion, along with the nearby mutation.

Human Mut Y homolo [MUTYH] (PDB ID: 8FAY) is involved in the excision of adenine inserted to complement 8-oxoguanine (Nuñez et al., 2018). MUTYH is a 546-aa-long protein. The N-terminal domain [NTD] encompasses a six-helix barrel with [4Fe–4S] cluster for metal binding, while the C-terminal domain undergoes crosstalk with AP:8-oxoG in DNA (Nakamura et al., 2021). The inter-domain connector [IDC] of MUTYH traverses through the DNA major grooves and coordinates the DNA repair/apoptosis process through an interaction with several effector proteins (Luncsford et al., 2010; Nakamura et al., 2021). The IDC region communicates with the Rad9–Rad1–Hus1 complex (9–1–1) and SIRT6 in response to oxidative DNA damage (Lim et al., 2015; Luncsford et al., 2010; Shi et al., 2006). The 4Fe–4S cluster with four conserved Cys moieties is essential for mismatch recognition and has a redox cofactor role (Bartels et al., 2017; Nuñez et al., 2018). The second putative metal [Zn+]-binding motif is located in the extended IDC region of mammalian MUTYH, which is constituted with three conserved Cys residues [ Cys 318, Cys 325, and Cys 328], and demonstrates control over adenine glycosylase activity (Bartels et al., 2017; Engstrom et al., 2014). Despite contradiction regarding the amino acid satisfying the fourth coordinate of Zn+, H72 came up with recent structure analyzed in comparison with mouse MUTYH (Nakamura et al., 2021) (Figure 8). Q310H substitution (rs3219489) has been identified among 40% OSCC patients under study. His310 is found in close vicinity of the Zn-binding motif in IDC and creates an opportunity for quenching the fourth coordinate of the metal ion (Figure 9). This possibility creates a significant effect on the overall conformation and fold of the MUTYH by distancing the N-terminal of the 6-helix barrel. No report about the correlation of Q310H with human carcinoma has been published yet.

### Association between SNPs and ineffectiveness of anticancer drugs/treatment

The atlas of cancer treatment revolves around the surgical procedure followed by adjuvant radiation therapy (aRT) and/or concurrent chemoradiation (CRT). The primary goal of radio-chemotherapy is to induce irreversible DNA damage in tumor/cancer cells that leads to cell death. This strategy relies on the inability of tumor cells to properly repair the DNA damage, which would otherwise allow them to survive and proliferate. While DNA repair mechanisms help prevent the onset of cancer in the normal condition, in established tumors, enhanced DNA repair capacity can contribute to tumor survival, allowing cancer cells to endure the damaging effects of radio-chemotherapy (Helleday et al., 2008; Wang et al., 2016). Radiation also triggers the formation of reactive oxygen species (ROS) and nitric oxide species (RNOS) that, in turn, induce inflammation and stress response, followed by the release of cytokines and chemokines (Bentzen, 2006; Zhao et al., 2007).Any patient exposed to radiotherapy certainly experiences the related toxicity, which can be minor to severe and extend for weeks to lifetime (Azria et al., 2008; Riballo et al., 1999; West and Barnett, 2011). Genetic variability plays a crucial role in how individuals from different ethnic background respond to various treatments. These variations can influence drug metabolism, efficacy, and potential toxicity, leading to different treatment outcomes (Kerns et al., 2015). Therefore, the future objective for cancer treatment should be oriented toward tailored chemo-radiation plans based on genetic determinants to minimize cancer/tumor progress.

**FIGURE 10:**
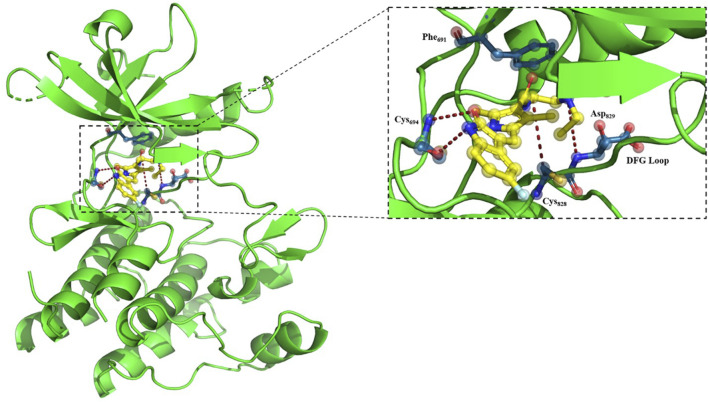
Docked conformation of sunitinib bound to the FLT3 tyrosine kinase domain (DFGin-active conformation of the kinase, PDB ID: 6JQR).

Accordingly, we analyzed the genetic inheritance of OSCC patients under study in correlation with possible toxicity due to chemo-radiotherapy. Our study highlights the FLT3 substitution at 227;interestingly, the same alteration was found to trigger toxicity against sunitinib treatment (Kim et al., 2013). Sunitinib binds with the active conformation of the FLT3 kinase (DFGin) through a specific interaction with C694 at the hinge region and D829 at the DFG motif (Acharya et al., 2022). T227M alteration may influence the monomeric–dimeric interconversion of FLT3 and affect the binding of sunitinib with the activated kinase domain with no effect on cancer cells, while, as a multitarget kinase inhibitor, it continues to work on receptor tyrosine kinases and hampers the normal physiological functions, resulting in toxicity. Figure 10 depicts the interaction details between FLT3 and sunitinib.

The XPC SNPs [rs2228000 and rs2228001] and their role are well-realized in case of cancers, but their association with post radio-chemotherapy adversity is yet to be fully understood. The radio-chemotherapy sensitivity of XPC [rs2228000 and rs2228001] for lung squamous cell carcinoma and head and neck cancer has been recently reported (Maćkowiak et al., 2024; Wang et al., 2016). Especially, in the case of rs2228001, polymorphism at the 939 position causes higher morbidity and adverse effects during and post-treatment (Maćkowiak et al., 2024). Being an important member of excision repair (BER), genetic alteration in XRCC1 [rs25487 and rs1799782] has a substantial effect on carcinoma progress and treatment itself. The effect of these SNPs on chemo-radio sensitivity is controversial and needs further studies. A recent meta-analysis correlates high XRCC1 expression with increased risk of minor treatment response and lesser chance of overall survival. It is further confirmed that rs25487 is unlikely to be toxic post-therapy for esophageal cancer, while it can trigger acute RT-induced adverse effects in head and neck cancer (Gong et al., 2021; Gu et al., 2015). Another meta-analysis concludes the Arg194Trp polymorphism is sensitive to platin-based drugs, while Arg399Gln has reduced sensitivity toward it in non-small-cell lung cancer in the Chinese population (Gu et al., 2015). *ERCC5* plays a pivotal role in NER, and hence, SNPs associated [rs1047768 and rs17655] with this protein are well-studied for their effect in inducing cancer. The connection of these SNPs, rs1047768 [His 46 Gln] and rs17655 [Asp1558 His, Asp1104 His], with chemo-radiotherapy-induced toxicity is still disputed. While meta-analysis on the Egyptian population demonstrated that these SNPs have no effect on the clinical outcome of platinum-based chemotherapy in NSCLC, another similar study on the Chinese population concluded that the C allele carriers of the *ERCC5* rs1047768 polymorphism are more sensitive to platinum-based chemotherapy (Abdalkhalek et al., 2022; Xu et al., 2018). The association of rs1801516 in ATM with cancer onset is well-realized, but its correlation with adverse outcomes post-radiotherapy is still inconclusive. While some publications refute any connectivity of rs1801516 with radiotoxicity, the meta-analysis on breast and prostate cancer postulates the correspondence between this gene alteration with an increased risk of radiation-induced normal tissue toxicity (Andreassen et al., 2016; Bensenane et al., 2024). Doxorubicin has been used extensively to treat various types of cancer as an antineoplastic agent. Apart from assisting in DNA damage through topoisomerase II, doxorubicin can stimulate ATM activation by S1981 phosphorylation and, hence, assist in the process of DNA double-strand repair directly and arrest the cell-cycle phase (Hublarova et al., 2010; Kurz et al., 2004). In addition to the activation of the ATM/P53 pathway, doxorubicin triggers the MAPK/NF-κβ survival pathway by interacting with PI3K family members, which may lead to drug resistance (Panta et al., 2004). Hence, the inhibition of ATM in combination with radiotherapy or chemotherapy would be considered a better treatment option, where DNA damage is the preferred pathway to trigger innate immunity (Dong et al., 2022; García et al., 2022).

## Conclusion

Overall, our study highlights the critical role of pharmacogenomics in advancing personalized cancer care, particularly in OSCC, which is a malignancy marked by poor prognosis and high therapeutic resistance. By integrating mutational profiling with structural and functional analysis, we identified key SNPs that may modulate drug response, DNA repair efficiency, and treatment outcomes. Notable variants in genes such as *FLT3, XRCC1, XPC, MUTYH*, and *ATM* were found to induce conformational alterations that could influence carcinogenesis, chemoresistance, and radiotoxicity. The novel discovery of *FLT3* T227M and *MUTYH* Q310H in OSCC patients from Southwest India further underscores the value of region-specific genomic data in shaping precision oncology strategies and improving therapeutic decision-making.

Despite the strengths of this integrative approach, the interpretations are dependent on *in silico* analysis, which requires further experimental validation. The proposed mechanistic hypotheses such as altered kinase activation and drug-binding efficiency in *FLT3* T227M or disrupted zinc coordination affecting MUTYH repair necessitate verification through cellular models, biochemical assays, and high-throughput structural assessment. Hence, future studies must include expanded cohorts to verify the clinical relevance, functional assay, and further refined genotype-based treatment recommendations. Incorporating genetic screening into routine cancer care, alongside continued structural validations with the development of pharmacogenomic resources, will accelerate the translation of these discoveries into evidence-based personalized therapy for OSCC patients.

## Data Availability

Publicly available datasets were analyzed in this study. This data can be found here: https://bcga.iitm.ac.in/cbioportal/study/summary?id=HNSCC_Yenepoya_2025.
